# Double and Triple Ionisation of Isocyanic Acid

**DOI:** 10.1038/s41598-020-59217-7

**Published:** 2020-02-10

**Authors:** J. H. D. Eland, R. J. Squibb, A. J. Sterling, M. Wallner, A. Hult Roos, J. Andersson, V. Axelsson, E. Johansson, A. Teichter, S. Stranges, B. Brunetti, J. M. Dyke, F. Duarte, R. Feifel

**Affiliations:** 1 Department of Chemistry, Physical and Theoretical Chemistry Laboratory, Oxford University, South Parks Road, Oxford, OX1 3QZ United Kingdom; 2 Department of Physics, University of Gothenburg, Origovägen 6B, SE-412 96, Gothenburg, Sweden; 3 Chemistry Research Laboratory, Oxford University, Mansfield Road, Oxford, OX1 3TA United Kingdom; 4 IOM-CNR Tasc, SS-14, Km 163.5 Area Science Park, Basovizza, 34149 Trieste, Italy; 5 Dipartimento di Chimica e Tecnologie del Farmaco, Universitá Sapienza, Rome, I-00185 Italy; 6 ISMN-CNR, c/o Dipartimento di Chimica, Universitá Sapienza, Rome, I-00185 Italy; 7 School of Chemistry, University of Southampton, Highfield, Southampton, SO17 1BJ United Kingdom

**Keywords:** Chemical physics, Electronic structure of atoms and molecules

## Abstract

Double and triple ionisation spectra of the reactive molecule isocyanic acid (HNCO) have been measured using multi-electron and ion coincidence techniques combined with synchrotron radiation and compared with high-level theoretical calculations. Vertical double ionisation at an energy of 32.8 ± 0.3 eV forms the ^3^A” ground state in which the HNCO^2+^ ion is long lived. The vertical triple ionisation energy is determined as 65 ± 1 eV. The core-valence double ionisation spectra resemble the valence photoelectron spectrum in form, and their main features can be understood on the basis of a simple and rather widely applicable Coulomb model based on the characteristics of the molecular orbitals from which electrons are removed. Characteristics of the most important dissociation channels are examined and discussed.

## Introduction

Because of the importance of isocyanic acid, HNCO, in terrestrial environments^1–3^ and the interstellar medium^4^, spectra of this molecule and its singly charged ion HNCO^+^ have been widely studied both experimentally and theoretically. A rather complete listing of the earlier spectroscopic work is given in a recent paper by Holzmeier *et al*.^5^ on its normal and resonant Auger spectra. The electronic and geometric structure of neutral HNCO and of its three most stable isomers have been calculated^6,7^, as has the structure of the singly positive ion^8,9^. The dynamics of fragmentation of its positive ions, both singly and doubly charged, were studied ∼30 years ago by mass-spectrometric methods^10,11^ and more recently by coincidence methods^8,12^. The states of the singly charged ions, seen in the photoelectron spectrum, could be correlated to the dissociation pathways^8^, but this was not possible for the doubly charged ions^12^ as no spectroscopic information on them existed at that time. We now report spectra of the double and triple ionisation of HNCO by single photon impact, obtained using a multi-electron coincidence technique combined with synchrotron radiation in the soft X-ray region.

The coincidence techniques used in the present work involve the detection and energy analysis of all the electrons or electrons and ions emitted in encounters between single high energy photons and single gas-phase molecules. They rely mainly on the use of a magnetic-bottle time-of-flight analyser^13^ for the electrons, and monochromatic photons from an electron storage ring or laboratory light source for ionisation.

## Results and Discussion

**Figure 1:**
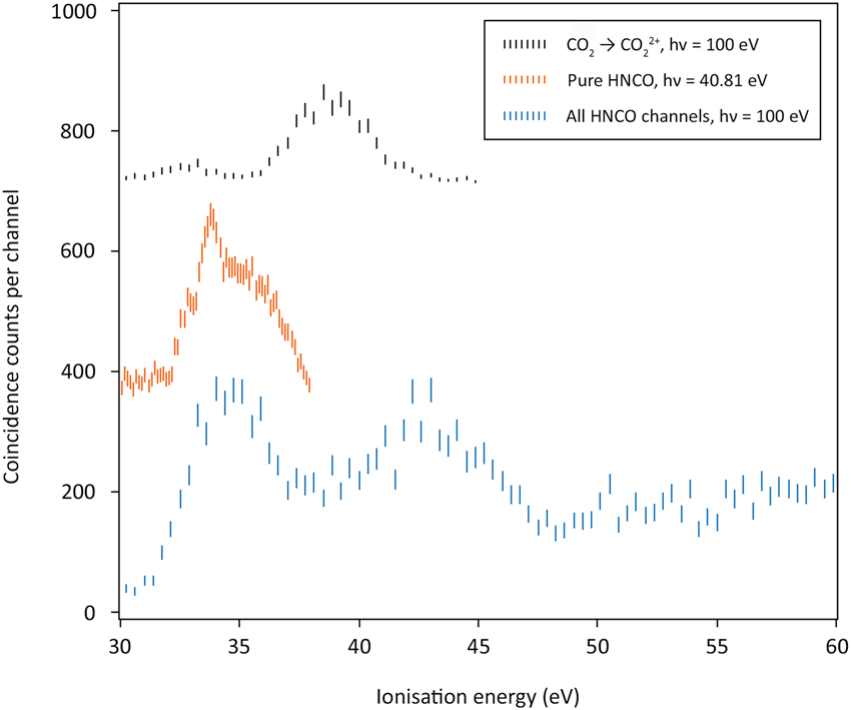
Valence double ionisation TOF-PEPECO spectra of HNCO. The lowest spectrum is a summation of all double ionisation channels attributed to HNCO made with coincident ion mass analysis at 100 eV. The uppermost spectrum shows the signal from CO_2_^2+^ at the same scale. The middle spectrum was measured in the laboratory at 40.81 eV photon energy with electrons only. Experimental points are represented by 2σ error bars. Resolution is estimated as 0.3 eV in the middle spectrum and 4 eV for the spectra at 100 eV photon energy.

Figure 1 shows valence double ionisation time-of-flight photoelectron-photoelectron coincidence (TOF-PEPECO) spectra of HNCO taken at 40.8 eV and 100 eV photon energy. Because some samples were contaminated by CO_2_ as explained in the experimental section, the 100 eV (lowest) spectrum of Fig. 1 is a summation of mass-resolved double ionisation channels attributed to the HNCO molecule. The uppermost spectrum, also at 100 eV, shows the signal from the CO_2_ contaminant, which if unrecognised would contribute a spurious peak at 39 eV ionisation energy. The central spectrum at 40.81 eV photon energy is an electron-only spectrum measured in the laboratory with a purer sample and better energy resolution. As this whole band lies below 37.34 eV, the lowest double ionisation energy of CO_2_^14^, it is entirely due to HNCO. The ionisation energy of the peak of the low-energy band at 33.8 eV is close to the energy of the lowest singlet state in the well-resolved normal Auger spectra of HNCO recorded by Holzmeier *et al*.^5^.

The overall spectrum at 100 eV bears a general resemblance to the calculated spectrum of singlet doubly ionised states from initial C1s^–1^ ionisation of Holzmeier *et al*.^5^, except for the lack of a distinct peak at 39 eV. The 40.8 eV spectrum has its peak at 33.8 eV and a distinct shoulder near at 32.8 eV. As the states produced by valence photoionisation are expected to include triplets as well as singlets, the shoulder at 32.8 eV in the better-resolved 40.8 eV spectrum which does not appear in the experimental or calculated Auger spectra^5^, probably represents ionisation to a triplet state of the dication. It is relevant to an interpretation of the spectrum to recall the orbital ordering in neutral HNCO (omitting the inner shells) which is

$$4{\rm{a}}\mbox{'}\,5{\rm{a}}\mbox{'}\,6{\rm{a}}\mbox{'}\,7{\rm{a}}\mbox{'}\,1{\rm{a}}\mbox{''}\,8{\rm{a}}\mbox{'}\,9{\rm{a}}\mbox{'}\,2{\rm{a}}\mbox{''}\ldots \ldots \ldots {}^{1}{\rm{A}}\mbox{''}$$

where all orbitals are doubly occupied, and the two outermost orbitals (2a” and 9a’) are the out-of-plane and in-plane components corresponding to the non-bonding π_g_ orbital of isoelectronic CO_2_. The next inner pair of orbitals (1a” and 8a’) correspond in the same way to the bonding π_u_ orbital of CO_2_.

**Figure 2:**
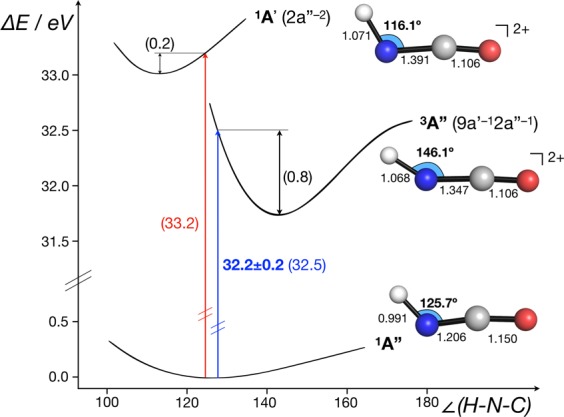
Double ionisation energies and key calculated structural data for HNCO (^**1**^**A”**) to the lowest energy triplet (^**3**^**A”**, 9a’^–1^2a”^–1^) and singlet (^**1**^**A’**, 2a”^−2^) dicationic states. Experimental ionisation energy in bold, calculated values at the CASSCF/MRCI level in parentheses. The ground state geometry was calculated at the CASSCF(8,6)/ano-TZVP level. Geometries of doubly ionised states were calculated at the CASSCF(6,6)/ano-TZVP level. Sections of the potential energy surfaces with respect to the ∠H-N-C angle were calculated at the level of theory used for optimisation of equilibrium structures (not to scale).

The difference in binding energy between the in-plane and out-of-plane non-bonding orbitals has been calculated as 0.8 eV^5^, in agreement with the vertical ionisation energy difference seen in the photoelectron spectrum^15^. In this present work, calculations at the CASSCF/MRCI (Complete active space self-consistent field/multi-reference configuration interaction) level of theory showed that the triplet state from 2a”^–1^ 9a’^–1^ ionisation lies 0.7 eV lower in energy than the first singlet dicationic state (2a” ^−2^). It is concluded therefore that the ground state of the doubly charged HNCO^2+^ ion is ^3^A” from 2a”^–1^9a’^–1^ ionisation and that this corresponds to the first feature in Fig. 1(a) with onset at 32.3 ± 0.2 eV and estimated peak at 32.8 ± 0.3 eV. At this level of theory, the vertical ionisation energy to this triplet state is calculated as 32.5 eV, in excellent agreement with experiments (Fig. 2).

**Figure 3:**
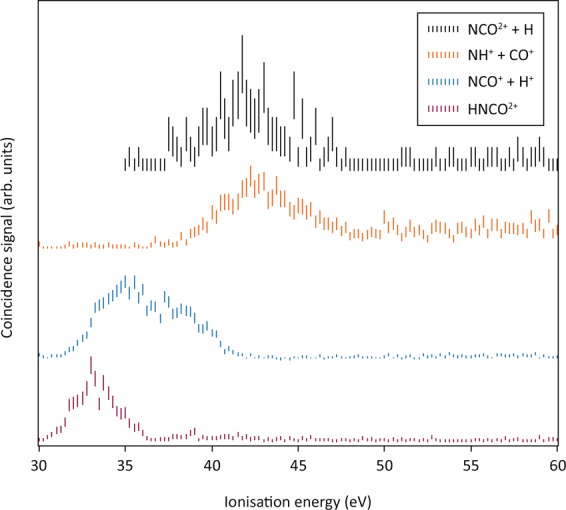
Double ionisation spectra coincident with the major dissociation channels of HNCO^2+^ measured at 100 eV photon energy. The four spectra are normalised to the same maximum amplitude for display because the true relative amplitudes were not directly measurable (see text). The energy resolution is estimated as about 4 eV at ionisation energies near 30 eV. Error bars show only the statistical uncertainty of the coincidence counts included. Three further channels, all of very low intensity, are listed in Table 1.

CASSCF/MRCI calculations predict the adiabatic double ionisation to be 0.8 eV lower in energy than the vertical, where the removal of a single electron from both the in-plane 9a’ and 2a” orbitals (^3^A”, Fig. 2) causes the molecule to change from a bent to a more linear equilibrium structure (∆[∠H–N–C] =  + 20.4°, Fig. 2). While the 9a’ orbital is formally non-bonding, we suggest that the origin of this distortion arises from the small contribution of the in-plane H1s to the molecular orbital^8^. As the 2a” orbital is almost entirely non-bonding, and removal of one electron from this orbital causes little change in geometric structure^8^, we expect the band from pure 2a”^−2^ ionisation to be narrow, without extended vibrational structure. Calculations at the CASSCF/MRCI level of theory predict the vertical double ionisation to the ^1^A’ (2a”^–2^) state to be only 0.2 eV greater than the adiabatic double ionisation to the same state (cf. Figure 2), suggestive of minimal structural distortion on double ionisation. The peak at 33.8 eV vertical ionisation energy is therefore assigned to this double ionisation. The second major peak in the 100 eV double ionisation spectrum at 38.5 eV, with a 1.3 eV half-width, is marginally narrower than the first. This is a normal width for a vibrationally extended single band and is almost at the apparatus-limited resolution of 1.2 eV in the 100 eV double photoionisation spectrum (60 eV electron kinetic energy). The first band in the 100 eV double ionisation spectra is clearly composite, most probably containing all four possible states from 9a’^−2^, 2a”^–1^9a’^–1^ and 2a”^–2^ ionisations. Similarly, the second major band at 38.5 eV must involve ionisation from the 8a’ and 1a” orbitals with closely spaced binding energies, as confirmed by the calculated singlet state energies^5^ and by sharpness of this band in the Auger spectra. It seems most probable that the long-lived HNCO^2+^ ions seen in the mass spectrum at 200 eV electron energy by Wang *et al*.^12^ are in the ground ^3^A” state created by 2a”^–1^9a’^–1^ ionisation. This is essentially confirmed by the ion-coincident double ionisation spectra shown in Fig. 3.

**Table 1:** Thresholds for HNCO^2+^ product formation after 100 eV photon impact.

Channel	Thermodynamiclimit (eV)	Observedonset (eV)
HNCO^2+^	(32.8)	32 ± 1
H^+^ + NCO^+^	30.2	33 ± 1
NH^+^ + CO^+^	31.3	39 ± 1.5
H + NCO^2+^	39.4*	39 ± 1.5
H^+^ + CO^+^ + N	34.7	36 ± 2
H^+^ + CN^+^ + O	38.1	43 ± 2
H^+^ + NO^+^ + C	34.5	39 ± 1.5

*Calculated value, see text.

To obtain the spectra for the ionic products (see Fig. 3) free from contamination by other ions, it was necessary to limit the ion flight-time ranges attributed to each, arbitrarily cutting down the total coincidence signals. For the ion pair products, the signals are also significantly weakened by loss of ions with initial sideways momentum, which hit the spectrometer walls or electrodes and so do not reach the detector. When the effect of these losses, estimated from the included TOF peak shapes is considered, the full intensities of the channels are estimated as HNCO^2+^, 78%, H^+^  + NCO^+^, 52% and NCO^2+^, 12% relative to NH^+^  + CO^+^ as 100% at 100 eV photon energy. These estimated intensities are in similar proportions to the cross-sections derived by Wang *et al*.^12^ from measurements of the same channels on electron impact ionisation at 200 eV. The spectrum coincident with undissociated HNCO^2+^ is consistent, in view of the estimated 3 eV energy resolution, with stability of this ion in a narrow energy range near or at the double ionisation onset. The appearance of NCO^2+^ at 39 ± 1.5 eV, combined with the dissociation energy of HNCO by H—NCO bond cleavage^16^ implies a double ionisation energy of NCO as 34.1 ± 1.5 eV, close to that of HNCO, as expected. Calculation at the CASSCF/MRCI level of theory confirms this, giving the double ionisation energy of NCO as 34.5 eV. This means that dissociation to H + NCO^++^ from the HNCO^++^ ground state can take place without any substantial kinetic energy release and no reverse activation energy in the pathway. For the H^+^  + NCO^+^ pair, the observed appearance energy near 33 eV implies kinetic energy release of about 5 eV, which is normal for such a charge separation forming ground state products. The appearance of the NH^+^  + CO^+^ pair, on the other hand, is delayed to about 39 eV, nearly 8 eV above its thermodynamic threshold. This implies the existence, confirmed later by theory, of an activation barrier to this bond breaking, as the excess is much larger than a kinetic energy release due to Coulomb repulsion from the initial inter-charge distance.

Because of spectral congestion, kinetic energy releases in the ion-pair production reactions could not be measured directly in these experiments. We return to a general discussion of the dissociation processes of multiply charged HNCO ions in a later section.

In view of the stability and abundance of an [H, N, C, O]^2+^ doubly charged ion, it is pertinent to ask if HNCO^2+^ or another isotopic form is the most stable. Earlier work of Morokuma’s group^9^ gives a relative energy ordering for the four most stable isomers of [H, N, C, O] (HNCO < HOCN < HCNO < HONC) for both the neutral and the singly-ionised states, calculated at the B3LYP/6-311 G(*d*, *p*) level of theory^9,17^. From calculations performed at the same level of theory in this present work, the triplet state is favoured over the singlet for each of the doubly-ionised isomers, as expected. The ordering of isomer energies is unchanged: HNCO < HOCN < HCNO < HOCN. We do note, however, that a substantial increase in static correlation in the doubly-ionised species may result in poor performance of the B3LYP functional for these systems. For example, for HNCO, the singlet-triplet splitting was calculated to be 1.58 eV, compared with 1.32 eV calculated at the CASSCF/MRCI level of theory.

### Core-valence (CV) spectra

**Figure 4:**
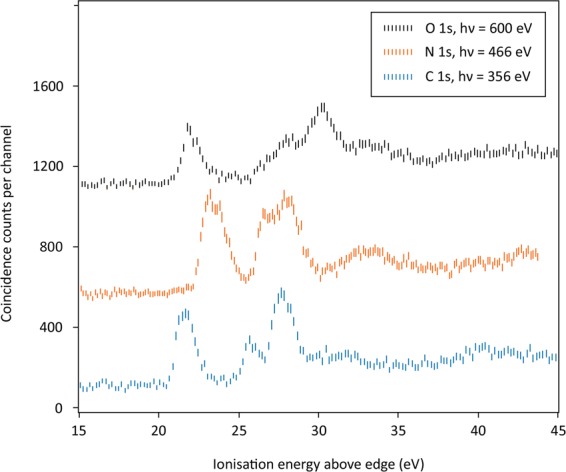
Core-valence spectra of HNCO above each of the edges, at the photon energies shown.

In a second form of double ionisation, one electron is removed from a valence orbital and another is removed from a core orbital, here the 1 s orbital of one of the C, N or O atoms. Because the core orbitals are remote in space and energy from the valence orbitals they hardly affect the molecular bonding, apart from the electrostatic influence of the localised core charges. As a result, core-valence double ionisation spectra are closely related to the photoelectron spectra from single valence electron ionisation. The three core-valence double ionisation spectra of HNCO in Fig. 4 illustrate this relationship.

**Figure 5:**
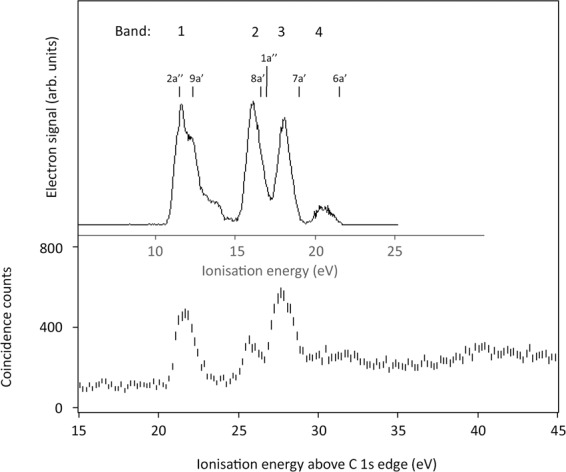
The C1s^−1^V^−1^ TOF-PEPECO spectrum (lower panel) of HNCO contrasted with a conventional photoelectron spectrum (upper panel) from single ionisation at 100 eV photon energy. Calculated binding energies of the first six orbitals^5,8^, shifted to fit to the first photoelectron band, are shown above, with identification of the four composite bands discussed in the text.

Each of the core-valence spectra in Fig. 4 has four main bands with spacings between them similar to the spacings of bands in the conventional photoelectron spectrum. Although these spectra have been taken without coincident mass analysis, comparison with the established core-valence spectrum of CO_2_^18^ shows no contamination in the spectra at the C1s or N1s edges. In the spectrum above the O1s edge only the weak shoulder at 27 eV and some of the intensity at 32 eV is probably due to CO_2_. Comparison with the photoelectron spectrum is further illustrated in Fig. 5, where the core-valence spectrum above the C1s edge is contrasted with a conventional photoelectron spectrum measured in the same apparatus at 100 eV photon energy, where the instrumental resolution is about the same.

Comparing Figs. 4 and 5, it can be seen that the numbers and spacings of bands in the two sorts of spectra (photoelectron and core-valence spectra) are similar. This indicates first of all that the singlet-triplet splittings of the core-valence states are relatively small, as also observed in the analysis of some other core-valence spectra^19,20^. To understand the shifts and relative intensities seen in Fig. 3 we propose a simple model, which should apply to molecules as big as HNCO or bigger. It is unlikely to work for small molecules such as diatomics, where effects related to spin-orbit and spin-spin coupling and other complications are more significant^21,22^. For such medium-sized molecules, we express the core-valence ionisation energy *IE*_*j*_ from each orbital *j* above the relevant edge as

$$I{E}_{j}-{E}_{edge}=I{P}_{j}+{e}^{2}/{r}_{12}$$

where *IP*_*j*_ is the valence orbital binding energy in the neutral molecule taken from the photoelectron spectrum and *e*^2^*/r*_12_ is a notional Coulomb repulsion energy between the charge in the delocalised molecular orbital and the localised core charge. Since the orbital ionisation energies are known from the photoelectron spectra^8,17^ the measured ionisation energies of each band above the related edge gives the notional Coulomb energy and apparent inter-charge distance *r*_12_. The general validity of this model is demonstrated by systematic changes in the notional Coulomb energy as a function of the size of several molecules. In molecules with just one heavy atom such as HCl, H_2_O or NH_3_ the Coulomb energy is between 12 and 16 eV. In CF_4_ it is about 8 eV, in SF_6_ and C_6_H_6_ it is about 5 eV and in C_60_ it falls to about 2 eV^18^. The apparent inter-charge distances r_12_ from the Coulomb energies correspond in every case to the approximate dimensions of the molecules. Within a single molecule, explanation of differences in the Coulomb shifts in ionisation from different valence orbitals calls for an extension of the model. To approach this, we consider the characters of the molecular orbitals, particularly the spatial distribution of their charge densities.

Because the in-plane and out-of-plane orbitals in HNCO, corresponding to each of π_g_ and π_u_ molecular orbitals in CO_2_, are unresolved in the single ionisation photoelectron spectrum recorded in this work, we group them and discuss the spectra in terms of four composite bands, 1 – 4, as shown in Fig. 5 and listed in Table 2.

**Table 2:** HNCO core-valence band Coulomb energies.

Band (IP/eV):		1 (12)	2 (15.5)	3(17.5)	4 (21)
Atom	Edge/eV	Coulomb energies (sh = shoulder)			
C	296.0	9.6	10.2	10.2	10.7
N	406.3	**11.4**	**11.3**(sh)	10.6	**12.2**
O	539.9	10.0	**11.8**	**12.5**	**12.5**

Larger values in bold.

A first observation is that in the CV spectrum of HNCO above the C edge, Fig. 5, the spacing of the four bands closely matches the spacing of the bands in the single ionisation photoelectron spectrum, whereas the spacings are markedly different when the core-hole is on N or O (see Fig. 4). The apparent Coulomb energies and inter-charge distances relevant to each band can be derived from comparison of the core-valence spectra with the photoelectron spectrum^8,15^. Apparent inter-charge distances for all four bands in the carbon-edge CV spectrum are about 1.4 Å, which is slightly larger than the C—N and C—O bond lengths (1.21 and 1.17 Å) in the neutral molecule^6–8,19^. Some bands in the N- and O- edge CV spectra exhibit larger apparent Coulomb energies and shorter inter-charge distances (bands 1 and 4 in the N-edge CV spectrum, bands 2, 3 and 4 in the O-edge CV spectrum). No band exhibits a longer apparent inter-charge distance than 1.4 Å, for example, none is near the overall length of the molecule of 2.5 Å. These characteristics must be related to the forms of the molecular orbitals, and as a rough guide to these, we can use the atomic orbital coefficients tabulated by Holzmeier *et al*.^5^ for the valence molecular orbitals of the neutral ground state molecule.

The summed squares of the orbital coefficients for the six orbitals that contribute to bands 1 to 4, Table 3, have characteristics that relate clearly to the CV spectra. First, no orbital (or π_g_/ π_u_ pair) has any strong concentration on the C atom. This is consistent with the close match between the CV spectrum above the C1s edge and the photoelectron spectrum. The greatest concentration of all (bold in Table 3) is of the π_g_ pair (band 1) on the N atom, which correlates with the large apparent Coulomb energy (11.5 eV, r_12_ = 1.25 Å) shown by this band when the core charge is on N. The next notable concentrations are of the orbitals for bands 2 and 3 on the O atom, again explaining the significant shifts of these bands to higher ionisation energy when the core charge is on O. For the N-edge CV spectrum, the orbitals corresponding to bands 2 and 3 have no strong concentration on N, while the orbital for band 4 is concentrated there, consistent with its larger apparent Coulomb energy. Overall, the qualitative agreements found in this way and demonstrated in the comparison between Tables 1 and 2 clearly show that the basic physics underlying the formation of the CV spectra is expressed in the Coulomb model.

**Table 3:** MO relative atom densities in HNCO from ref. ^5^.

Band:	1	2	3	4
C	0.02	0.21	0.14	0.13
N	**0.62**	0.12	0.06	**0.44**
O	0.36	**0.39**	**0.84**	0.10

Largest density in each orbital or combination in bold. *To make this table, atomic orbital coefficients from ref. ^5^ have been squared without normalisation, summed in pairs then divided by two for groups 1 and 2.

This simple molecular orbital and Coulomb model also works in other cases. In CO_2_, whose outermost (π_g_) orbital is located only on the O atoms, the first band in the CV spectra^18^ is shifted to higher energy when the core hole is on an O atom than when it is on the C atom. In the CV spectrum of CF_4_ with a hole in C1s, the bands from orbitals with C—F bonding character are shifted to higher energy than those with pure F lone-pair character. Similarly, for acetaldehyde, whose HOMO is located strongly on the O atom, the lowest energy CV band is at considerably higher energy when the core hole is on the O atom than when it is on either of the C atoms.

### Triple ionisation

**Figure 6:**
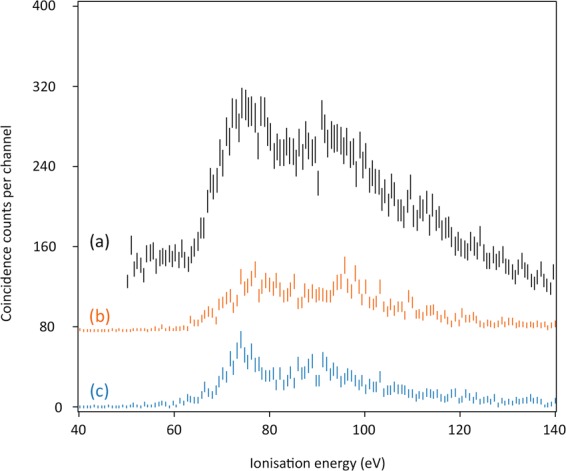
Triple ionisation (PEPEPECO) spectra of HNCO: (a) from double Auger decay of the C1s hole state at 296.0 eV using 316 eV photon energy; (b) from single Auger decay of the lowest energy core-valence doubly ionised state at ca 318 eV (band 1); (c) from single Auger decay of the core-valence state at about 324 eV (band 3). Photon energy for the CV spectra was 356 eV.

HNCO can be triply ionised directly at photon energies below and above all the inner shell ionisation energies, by double Auger decay from the three 1s^−1^ hole states, or by single Auger decay from the doubly ionised CV states discussed above. In practice, the triple ionisation cross-section at photon energies below the inner shells is too small to give a significant signal above background in the present experiments. Of the other possibilities, only Auger decay from the C1s hole state and from the associated CV states, giving Auger electron energies of about 200 to 250 eV, offers useful electron energy resolution with the present apparatus. Three spectra are presented in Fig. 6.

The three triple ionisation spectra in Fig. 6 are similar in form, with onset at about 65 eV and two broad peaks, the first near 75 eV and a second 20 or 25 eV higher in energy. Because of the likelihood of nuclear motion in an intermediate state (core-hole or valence hole(s) for spectrum (a), core valence states for (b) and (c)) the onset energies cannot be assumed to represent pure vertical transitions. However, a calculation at the CASSCF/MRCI level gives the vertical triple ionisation energy as 64.8 eV, agreeing with the onsets of all three forms of the triple ionisation spectra in Fig. 6 at 65 ± 1 eV. This is considerably lower than the triple ionisation energy of CO_2_, previously determined by a similar method as 74 ± 0.5 eV^20^. The lack of fine detail in the triple ionisation spectra is caused partly by the instrumental resolution (ca 4 eV) but also by the expected congestion of electronic states. The removal of three electrons from the six outer valence orbitals can give rise to 55 electronic states, 35 doublets and 20 quartets, within an estimated 40 eV energy range. All are likely to be dissociative, giving broad Franck-Condon envelopes.

### HNCO di- and tri-cation dissociation dynamics

The pathways followed in fragmentation of nascent HNCO^2+^ and HNCO^3+^ and their partial cross-sections were determined by Wang *et al*.^12^ in their covariance analysis of electron-impact-induced dissociative ionisation. The present results show that photon impact at 100 eV photon energy produces many of the same products as does 200 eV electron impact, but with different relative abundances. Neither we nor Wang *et al*. have measured the kinetic energy releases in the different channels, so we cannot relate different channels directly to different elements of the spectra. It is nevertheless useful to compare the thermodynamic thresholds for the different channels with the experimental onsets of different product channels, with the usual magnitudes of kinetic energy releases in mind. The thresholds for formation of ground-state products determined from the tabulation of Lias *et al*.^16^ are listed in Table 1 for the dication dissociation pathways clearly identified in our data. All these channels are also listed by Wang *et al*.^12^. The appearance energies have wide error limits because of the low (ca 3 eV) resolution in the mass-resolved data. The last channel listed (H^+^  + NO^+^  + C) is particularly interesting if these products really come from a substantial rearrangement of the HNCO^2+^ ion. But we cannot entirely exclude the possibility that our samples contain a small proportion of fulminic acid (HCNO) as well as isocyanic acid.

**Figure 7:**
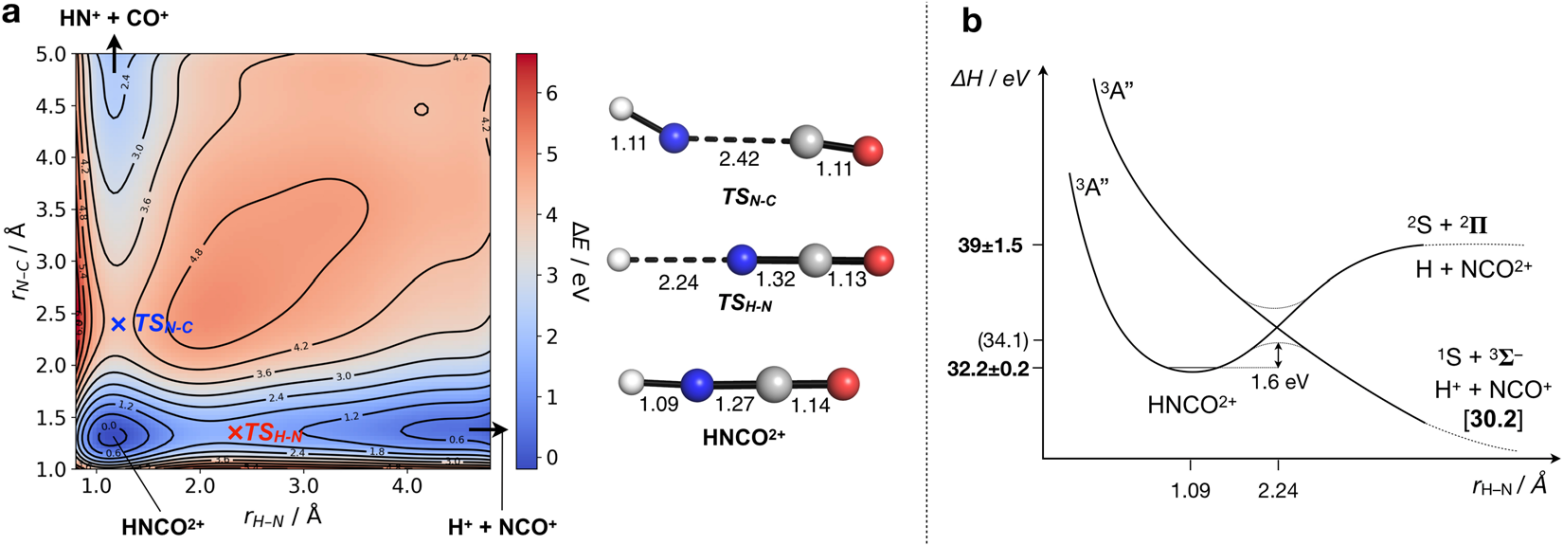
(**a**) Potential energy surface (left) and stationary points (right) for HNCO^2+^ in its triplet ground state as a function of the H–N and N–C bond lengths. (**b**) Schematic diabatic (HNCO^2+^→H + NCO^2+^) and adiabatic (HNCO^2+^→H^+^ + NCO^+^) potential energy surfaces for the stretching of the H–N bond of HNCO^2+^. Experimental (calculated) values in bold (parentheses), and thermodynamic limit in square brackets. All calculations carried out at the PBE0/def2-TZVP level, bond lengths in Å, and energies in eV.

From the presence of HNCO^2+^ ions in the mass spectrum and their appearance within a narrow energy range near the lowest double ionisation energy (cf. Figure 3 and Table 1), it is clear that the dication is stable or metastable in its ground state. Wang *et al*. observed a characteristic metastable signature for the dissociation to H^+^  + NCO^+^ in electron impact double ionisation. This means that there must be a barrier to dissociation towards H^+^  + NCO^+^, the pathway which takes over from persistence of the doubly-charged parent ion within about 1 eV of the dication ground state formation. These observations are confirmed by a calculation of the ground state potential energy surface for H—N and HN—CO bond extensions using DFT (density functional theory) at the PBE0/def2-TZVP level^23,24^, Fig. 7. The barrier to H—N bond cleavage is calculated at this level of theory as 1.6 eV, slightly higher than estimated experimentally. The barrier to HN—CO cleavage is calculated as 3.9 eV, in excellent agreement with the observed appearance energy.

The barrier to H—NCO bond breakage evidently arises from an avoided crossing between the diabatic surface leading to H + NCO^++^ and the adiabatic surface leading to charge separation. The lifetime for proton tunnelling through the barrier from at least one vibrational level is long enough (≈μs) for mass-spectroscopic observation and longer-lived levels may exist within the potential well. Both of the observed two-body dissociation pathways of HNCO^2+^ are spin-allowed, H^+^(^1^Σ^+^) + NCO^+^(^3^Σ^−^) and NH^+^(^2^Π) + CO^+^(^2^Σ^+^) and correspond to simple bond breakages. The barrier to the charge separating HN—CO bond breakage can be seen as arising from the orbital rearrangement necessary to produce CO^+^ in its ^2^Σ^+^ ground state rather than in the ^2^Π state which would correlate directly with the CO moiety in HNCO^++^. No strong signal due to intra-molecular rearrangement is observed, in contrast to the situation in the singly-charged ion^8^.

In triple ionisation, the thermodynamic fragmentation thresholds are all well below the energy range of states populated in the spectra of Fig. 6. If a representative kinetic energy release of 10 eV is added to the thermodynamic thresholds the estimated appearance energies are all lower than or within the low energy band of the triple ionisation spectrum, so the absence of a detected HNCO^3+^ ion^11^ is unsurprising. Three-body fragmentations occur at the lowest energies, but four-body decays with complete atomisation of the molecule are also possible for the majority of the states seen to be populated in Fig. 6.

## Conclusions

The valence double ionisation spectrum of HNCO, representing a showcase of the first of a class of reactive molecules investigated with the TOF-PEPECO technique, has identified and located the lowest electronic states of the dication. Electron-ion coincidence spectra of the low energy dissociation products, combined with calculation of the ground state potential energy surface show that only a few bound vibrational levels persist below the barrier to the first charge separation. This suggests that in astrophysical environments double ionisation by cosmic ray or EUV (extreme ultraviolet) impact will destroy the molecule. Core-valence double ionisation, while probably irrelevant to astrophysics, provides an interesting testing ground for simple physical theory. We show that a simple Coulomb model explains the main features of the core-valence spectra of HNCO, and by implication those of other compounds. A first triple ionisation spectrum of HNCO is also reported.

## Methods

### Experimental

Experiments were carried out at beamline UE52/SGM of the electron storage ring BESSY-II at the Helmholtz Zentrum Berlin when the ring was operated in single-bunch mode. Because the period of 800 ns between bunches in this mode is much shorter than the flight times of low energy electrons (up to 5000 ns) or of ions, a mechanical chopper^25^, synchronised to the ring pulses, was used to increase the inter-pulse period to about 12 μs in the source region of the magnetic bottle. At that point the light pulses intersect an effusive jet of the target gas from a hollow needle in the divergent field (ca 1 kG) of a permanent magnet, which directs almost all the emitted photoelectrons towards a distant detector. The 2 m long flight path is surrounded by a solenoid whose field lines guide the electrons to the microchannel plate detector where their arrival times relative to the light pulses are registered. Flight times are converted to electron kinetic energies with the help of calibration using well-known photoelectron and Auger electron energies. The energy resolution is limited mainly by imperfect parallelism of the electron trajectories and, for these experiments, could be expressed as a numerical ratio E/ΔE ≈ 50. For experiments in the laboratory the same electron spectrometer was used, but the photon source was a pulsed discharge in low-pressure He followed by a toroidal-grating monochromator^26^, providing 40.8 eV photons from the HeIIα atomic emission line. To examine the dissociations of HNCO^2+^ ions the same magnetic bottle was augmented with an in-line time-of-flight mass selector, which has been fully described before^27^. In brief, a pulsed ion draw-out field is applied to the source region once all electrons have escaped into the field-free flight tube. Ions are accelerated towards a microchannel plate detector by fields which impose the time-focussing conditions. Because a less intense divergent magnetic field is used in these electron-ion experiments compared to that used in the electron-only experiments, the electron resolution E/ΔE under these conditions is about 20 while the mass resolution (FWHM) is about 50.

Isocyanic acid was prepared by the reaction between potassium cyanate and an excess of molten stearic acid at 86 C. The reagents were finely ground and scrupulously dried over P_2_O_5_ in vacuo for several days before use. The raw reaction products were condensed in a liquid nitrogen (LN_2_) trap, then vacuum-distilled into a trap cooled by a solid CO_2_-acetone bath before repeated trap-to-trap distillations and final short-period storage at LN_2_ temperature for admission to the apparatus. Despite the attempted purification a small but significant contamination by residual CO_2_ from decarboxylation of stearic acid remained and was present to a variable extent in different experimental runs. Its effect could be eliminated in runs with coincident mass analysis, and could be recognised by comparison with known CO_2_ spectra in evaluation of the electron-only spectra.

### Computational

Calculations were carried out using the ORCA suite of programs (version 4.1.1)^28^. CASSCF (Complete Active Space Self-Consistent Field) calculations for HNCO, HNCO^2+^ and HNCO^3+^ were run at the CASSCF(8,6)/ano-TZVP, CAS(6,6)/ano-TZVP and CAS(5,6)/ano-TZVP levels of theory, respectively^29^. These active spaces incorporate 8 (neutral), 6 (doubly ionised) and 5 (triply ionised) electrons distributed in the bonding, non-bonding and antibonding orbital configurations of both the in-plane and out-of-plane π-systems (1a” 8a’ 9a’ 2a”10a’3a”). Structures were confirmed to be local minima by the absence of imaginary frequencies upon calculation of the Hessian. Dynamic correlation was incorporated with the multi-reference configuration interaction (MRCI) method including single and double excitations^30^. DFT calculations of isomers of [H, N, C, O] were carried out at the B3LYP/6-311 G(*d*,*p*) level of theory for direct comparison with previous studies^9,28^. The potential energy surface calculations for the dissociation of HNCO^2+^ were calculated at the PBE0/def2-TZVP level of theory and consist of a 400 point (20 × 20) grid of H–N and N–C bond lengths, from 0.8–4.8 and 1.0–5.0 Å, respectively. Transition states were characterised by the appearance of a single imaginary frequency upon calculation of the Hessian, corresponding to bond cleavage under study.

## Data Availability

The datasets generated during and/or analysed during the current study are available from the corresponding author on reasonable request.

## References

[1] Miller JA, Bowman CT (1991). Kinetic modelling of the reduction of nitric oxide in combustion products by isocyanic acid. Int. J. Chem. Kinetics.

[2] Karlsson D, Dalene M, Skarping G, Marand A (2001). Determination of isocyanic acid in air. J. Environ. Monit..

[3] Leslie MD (2019). Isocyanic acid (HNCO) and its fate in the atmosphere: a review. Environ. Sci. Processes Impacts.

[4] Quan D (2010). Gas-grain modeling of isocyanic acid (HNCO), cyanic acid (HOCN), fulminic acid (HCNO), and isofulminic acid (HONC) in assorted interstellar environments. Astrophys. J..

[5] Holzmeier F (2018). Normal and resonant Auger spectroscopy of ioscyanic acid, HNCO. J. Chem. Phys..

[6] East ALL, Johnson CS, Allen WD (1993). Characterization of the X̃ ^1^A’ state of isocyanic acid. J. Chem. Phys..

[7] Mladenović M, Elhiyani M, Lewerenz M (2009). Electric and magnetic properties of the four most stable CHNO isomers from *ab initio* CCSD(T) studies. J. Chem. Phys..

[8] Wilsey S, Thomas SE, Eland JHD (2000). An experimental and theoretical study of the HNCO^+^ ion. Chem. Phys..

[9] Luna A, Mebel AM, Morokuma KJ (1996). Density functional study of the global potential energy surfaces of the [H, C, N, O]^+^ system in doublet and quartet states. Chem. Phys..

[10] Hop CECA (1987). [HCNO]^+^⋅, [HNCO]^+^⋅ and their neutral counterparts studied by mass spectrometry. Rapid Commun. Mass Spectrom..

[11] Rowland CG, Eland JHD, Danby CJ (1969). Kinetic energy distributions of fragment ions in the mass spectrum of isocyanic acid. Int. J. Mass Spectrom. Ion Phys..

[12] Wang P (2004). Dissociation of multiply ionized isocyanic acid through electron impact. J. Chem. Phys..

[13] Hult Roos A (2017). Valence double ionization electron spectra of CH_3_F, CH_3_Cl and CH_3_I. Chem. Phys..

[14] Slattery AE, Field T (2005). Spectroscopy and metastability of CO_2_^2+^ molecular ions. J. Chem. Phys..

[15] Eland JHD (1970). The Photoelectron Spectra of Isocyanic Acid and Related Compounds. Phil. Trans. Roy. Soc. London A.

[16] Lias, S.G. *et al*. Gas-phase ion and neutral thermochemistry. *J. Phys. Chem. Reference Data* **17**, Supplement 1 (1988).

[17] Mebel AM, Luna A, Lin C, Morokuma KA (1996). A density functional study of the global potential energy surfaces of the [H,C,N,O] system in singlet and triplet states. J. Chem. Phys..

[18] Eland, J.H.D. & Feifel, R. Double Photoionisation Spectra of Molecules. Oxford University Press, pp. 162 et seq. (2018).

[19] Yamada KJ (1980). Molecular structure and centrifugal distortion constants of isocyanic acid from the microwave, millimeter wave, and far-infrared spectra. Mol. Spectrosc..

[20] Eland JHD (2011). Triple ionization of CO_2_ by valence and inner shell photoionization. J. Chem. Phys..

[21] Hikosaka Y (2007). Multielectron coincidence spectroscopy for core-valence doubly ionized states of CO. J. Chem. Phys..

[22] Valiev RR (2017). Optimization of core-valence states of molecules. Mol. Phys..

[23] Adamo C, Barone V (1999). Toward reliable density functional methods without adjustable parameters: The PBE0 model. J. Chem. Phys..

[24] Weigend F, Ahlrichs R (2005). Balanced basis sets of split valence, triple zeta valence and quadruple zeta valence quality for H to Rn: Design and assessment of accuracy. Phys. Chem. Chem. Phys..

[25] Plogmaker S (2012). Versatile high-repetition-rate phase-locked chopper system for fast timing experiments in the vacuum ultraviolet and x-ray spectral region. Rev. Sci. Instrum..

[26] Eland JHD (2009). Dynamics of Double Photoionization in Molecules and Atoms. Adv. Chem. Phys..

[27] Eland JHD, Feifel R (2006). Double ionisation of ICN and BrCN studied by a new photoelectron-photoion coincidence technique. Chem. Phys..

[28] Neese F (2018). Software update: the ORCA program system, version 4.0. Wiley Interdiscip. Rev. Comput. Mol. Sci..

[29] Neese F, Valeev EF (2011). Revisiting the Atomic Natural Orbital Approach for Basis Sets: Robust Systematic Basis Sets for Explicitly Correlated and Conventional Correlated ab initio Methods?. J. Chem. Theory Comput..

[30] Streit L (2011). Double ionization energies of HCl, HBr, Cl_2_ and Br_2_ molecules: An MRCI study. Chem. Phys. Lett..

